# 758. Increased Prevalence of *Clostridioides difficile* Infection During the COVID-19 Pandemic Among Hospitalized Veterans in South Texas, USA

**DOI:** 10.1093/ofid/ofab466.955

**Published:** 2021-12-04

**Authors:** Eric H Young, Erica Beck, Delvina Ford, Julieta Madrid-Morales, Ann Hoffman, Jose Cadena-Zuluaga, Alexa Frei, Kelly R Reveles

**Affiliations:** 1 University of Texas at Austin, San Antonio, TX; 2 South Texas Veterans Health Care System, San Antonio, Texas; 3 South Texas Veterans Health Care System, Audie L. Murphy Division, San Antonio, TX; 4 University of Texas health and science center San Antonio, Audie L. Murphy VA Medical Center, San Antonio, Texas; 5 University of Texas at Austin College of Phramacy, San Antonio, Texas

## Abstract

**Background:**

*Clostridioides difficile* infection (CDI) continues to be a major global public health concern, particularly during the ongoing SARS-CoV-2 coronavirus disease 2019 (COVID-19) pandemic. Despite new social distancing guidelines and enhanced infection control procedures (e.g., masking, hand hygiene) being implemented since the beginning of COVID-19, little evidence indicates whether these changes have influenced the prevalence of CDI hospitalizations. This study aims to measure CDI prevalence before and during the COVID-19 pandemic in a local cohort of U.S. Veterans.

**Methods:**

This was a cross-sectional study of all Veterans presenting to the South Texas Veterans Health Care System in San Antonio, Texas from Jan 1, 2019 to Apr 30, 2021. Monthly laboratory confirmed CDI events were collected overall and categorized as the following: hospital-onset, healthcare facility-associated (HO-HCFA-CDI), community-onset, healthcare facility-associated CDI (CO-HCFA-CDI), and community-associated CDI (CA-CDI). Monthly confirmed COVID-19 cases were also collected. CDI prevalence was calculated as CDI events per 10,000 bed days of care (BDOC) and was compared between pre-pandemic (Jan 2019-Feb 2020) and pandemic (Mar 2020-Apr 2021) periods.

**Results:**

A total of 285 CDI events, 920 COVID-19 cases, and 104,220 BDOC were included in this study. The overall CDI rate increased from 20.33 per 10,000 BDOC pre-pandemic to 34.51 per 10,000 during the pandemic (p< 0.0001). This was driven primarily by a rise in CO-HCFA-CDI rates (0.95 vs 2.52 per 10,000 BDOC; p< 0.0001) during the pandemic, followed by increases in CA-CDI (15.58 vs. 18.61 per 10,000 BDOC; p< 0.0001) and HO-HCFA-CDI (2.66 vs. 5.43 per 10,000 BDOC; p< 0.0001). Lastly, CDI rates have tripled since the start of the pandemic (March-Apr 2020) compared to the current year (March-Apr 2021) (14.69 vs. 43.76 per 10,000 BDOC).

**Conclusion:**

Overall, CDI prevalence increased during the COVID-19 pandemic, driven mostly by an increase in CO-HCFA-CDI. As COVID-19 rates increased, CDI rates also increased, likely due to greater healthcare exposures and antibiotic use. Continued surveillance of COVID-19 and CDI is warranted to further decrease infection rates

**Disclosures:**

**All Authors**: No reported disclosures

